# Pro-inflammatory response of human iPSC-derived intestinal epithelial monolayers towards microbial toxins LPS and nigericin

**DOI:** 10.1007/s00204-025-04215-9

**Published:** 2025-10-08

**Authors:** Germaine Aalderink, Hugo Brouwer, Jingxuan Wang, Aafke W. F. Janssen, Meike van der Zande, Coen Govers, Tamara Hoppenbrouwers, Hans Bouwmeester, Mathias Busch

**Affiliations:** 1 Division of Toxicology, Wageningen University and Research, Wageningen, The Netherlands; 2 Wageningen Food Safety Research, Wageningen University and Research, Wageningen, The Netherlands; 3 Division of Cell Biology and Immunology, Wageningen University and Research, Wageningen, The Netherlands; 4 Wageningen Food and Biobased Research, Wageningen University and Research, Wageningen, The Netherlands; 5 Food Quality and Design, Wageningen University and Research, Wageningen, The Netherlands

**Keywords:** Gastrointestinal tract, Stem cells, Caco-2, Intestinal barrier function, Cytokines

## Abstract

The intestinal epithelium forms a selective barrier between the intestinal lumen and the subepithelial layer. Intestinal epithelium plays a critical role in initiating inflammatory tissue responses in vivo, which remains challenging to emulate in vitro. Caco-2 cells are commonly used models of the intestinal epithelium, but lack crucial receptors and pathways associated with pro-inflammatory reactions. Human-induced pluripotent stem cell (iPSC)-based in vitro models are assumed to provide a system that better emulates in vivo responses. This study evaluated the inflammatory response of iPSC-derived intestinal epithelial cells (IEC) and Caco-2-derived intestinal epithelial cells to the microbial toxins lipopolysaccharide (LPS) and nigericin. Here, iPSCs were differentiated towards enterocyte, goblet- and Paneth-like cells without using three-dimensional culture techniques. The formed monolayer barriers were exposed to a combination of 0–100 µM nigericin and 100 ng/mL LPS on either the apical or basolateral side. The treatment-induced expression of cytokine genes and cytokine secretion were compared between the iPSC-derived cell model and differentiated Caco-2 cell layers. Nigericin exposure in combination with LPS significantly reduced transepithelial electrical resistance in the iPSC-derived model, and resulted in a tenfold increased secretion of the pro-inflammatory cytokines interleukin (IL)-6, IL-8, and tumor necrosis factor-alpha compared to the negative control. A similar increase was observed for the mRNA expression of these cytokines. No significant effect on TEER, cytokine secretion, or mRNA expression was observed in the Caco-2 model. Overall, this study shows that iPSC-IECs are a more sensitive model compared to Caco-2 to emulate inflammatory perturbations of the human intestinal epithelium.

**Supplementary Information:**

The online version contains supplementary material available at 10.1007/s00204-025-04215-9.

## Introduction

The human intestinal epithelium consists of a single-cell layer that separates the lumen containing the food-chyme and intestinal microbiome from the underlying submucosal tissues. The intestinal barrier protects the host from potentially harmful microbial and fungal species while being a selective barrier to nutrients and food-borne chemicals (Ménard et al. 2010). Importantly, the intestinal epithelium dynamically interacts with intestinal microbiome metabolites and submucosal cells via paracrine signaling and selective paracellular, and/or transcellular transport of the metabolites (Groschwitz and Hogan 2009). Proper barrier integrity and a physiological balance in epithelial cell pro-inflammatory and anti-inflammatory responses are key to maintaining a healthy gut physiology (McGuckin et al. 2009; Peterson and Artis 2014).

The intestinal epithelial cell (IEC) layer comprises various specialized cell types such as enterocytes, goblet cells, Paneth cells, M cells, and enteroendocrine cells. IECs are replenished approximately every 3–5 days (Darwich et al. 2014; van der Flier and Clevers 2009) through the proliferation, migration, and differentiation of LGR5 + intestinal stem cells that reside in the intestinal crypt (Barker et al. 2007). Each type of IEC has a specific function that is essential for maintaining intestinal homeostasis. Enterocytes comprise 70% of the epithelial lining and are primarily responsible for absorbing nutrients and food-borne chemicals from digested foods (Wang et al. 2019). Enterocytes, together with the other IEC types in the gut epithelium, are connected via a network of epithelial tight junction proteins that coordinate the paracellular permeability between IECs (Lee 2015). Goblet cells are the second most abundant cell type and secrete mucin glycoproteins, such as Mucin 2 (MUC2), to form a highly viscous, yet porous, mucus layer on top of the IEC layer (Kim and Ho 2010). The mucus layer serves as a lubricant for the transport of chyme and protects the epithelial layer from luminal contents (Johansson et al. 2008). It has been shown that impaired mucus secretion due to *Muc2* deficiency results in elevated levels of pro-inflammatory cytokines secreted by IECs, underpinning the importance of an appropriate mucus layer in immunotolerance (Johansson et al. 2008; Van der Sluis et al. 2006). Paneth cells reside in the crypts of the epithelial lining and secrete antimicrobial peptides in the mucus layer, which prevents the undesired infiltration of bacteria (Bevins and Salzman 2011; Dupont et al. 2014; Sato et al. 2009). Paneth cells, along with goblet cells and enterocytes, form a major component of the innate immune system of the small intestine and help regulate intestinal inflammation (Dupont et al. 2014). In addition, enteroendocrine cells detect the metabolites of commensal bacteria and release peptide hormones and cytokines to recruit immune cells and modulate their response (Worthington 2015). The so-called M cells selectively endocytose mainly particulates like bacteria to present them to macrophages and lymphocytes in the *lamina propria* on the basolateral side of the IEC layers (Wang et al. 2014). When the intestine is in homeostasis, host-borne microbial agents from the lumen of the gastrointestinal tract induce an anti-inflammatory state, which promotes immune tolerance in the intestinal tissue (Lin and Zhang 2017; Zhang et al. 2015).

Imbalances in gut microbiota can alter the epithelial immune response towards an inflamed state (Zhang et al. 2015). For example, luminal overgrowth of pathogenic bacteria can lead to an increased presence of a variety of endotoxins, including bacterial lipopolysaccharides (LPS), which can negatively affect the intestinal epithelial barrier (Ivanov et al. 2010). LPS interacts with the toll-like receptor 4 (TLR4) on IECs (Price et al. 2018), resulting in the secretion of pro-inflammatory cytokines and increased susceptibility to inflammatory diseases (Stephens and von der Weid 2020). Pro-inflammatory cytokines induce downregulation of tight junctions, most notably zonula occludens-1 (ZO-1), resulting in a subsequent decline in barrier integrity (Chelakkot et al. 2018). Loss of intestinal barrier integrity and long-term stimulation of pro-inflammatory pathways can result in chronic inflammation, leading to the development of inflammatory bowel diseases (IBD), such as Crohn’s disease (Dignass et al. 2012; Van Assche et al. 2010) and ulcerative colitis (Gonzalez-Licea and Yardley 1966; McGuckin et al. 2009). Emulating the response of epithelial cells to intestinal pathogens and their metabolites aids our understanding of intestinal homeostasis and disease development and progression (Macedo et al. 2023). To study the mechanisms of intestinal pathogens, it is essential to evaluate the inflammatory signaling capacity of appropriate intestinal epithelial models in vitro.

Several in vitro models of the intestinal epithelium have been used to replicate the complexity of intestinal inflammation to elucidate the pathogenesis of IBD (Macedo et al. 2023). The immortalized Caco-2 cell line is most often chosen as the preferred cell line due to its ability to spontaneously differentiate to an enterocyte-like phenotype after reaching confluency (Pinto et al. 1983). Caco-2 cells are epithelial cells derived from colon tissue of a colorectal adenocarcinoma, and their differentiation is marked by the transition of colonocyte markers to enterocyte markers between days 3–18 post-confluence (Engle et al. 1998), resembling the gene expression pattern of small intestinal enterocytes (Tremblay et al. 2006). However, differentiated Caco-2 cells lack important receptors involved in crucial pro-inflammatory pathways, such as TLR4 and IL1R (Böcker et al. 2003), and the cellular diversity required to model the interaction with immune cells in the intestinal epithelial layer in vivo. Several in vitro models are described to complement Caco-2 cells with additional immunocompetent cell lines, such as macrophage-like THP-1 (Kämpfer et al. 2017), dendritic cell-like MUTZ-3 (Susewind et al. 2016) or primary monocyte-derived macrophages and dendritic cells, to be able to detect inflammation-related responses (Lehner et al. 2020). However, the intestinal epithelium itself is a key player in pro-inflammatory responses and might even have opposite roles of typical immune cells in pathways that are involved in intestinal inflammation (Busch et al. 2022). To investigate the mechanisms underlying intestinal inflammation, such as that induced by pathogens, in vitro, it is essential to assess the inflammatory signaling capacity of appropriate intestinal epithelial models.

Primary microtissues would provide a relevant cell model, but their applicability is limited due to their restricted expansion, limited availability, and low expression of transporters compared to in vivo tissue (Ayehunie et al. 2018; Janssen et al. 2024a, b; Macedo et al. 2023). Adult stem cells, isolated from primary intestinal material, can be grown for extended periods when cultured in an extracellular matrix to form self-organizing three-dimensional intestinal organoids (Pleguezuelos-Manzano et al. 2020). The cellular composition within these organoids is modulated by a combination of growth factors (Beumer and Clevers 2021; He et al. 2022). However, intestinal organoids, derived from either adult stem cells or induced-pluripotent stem cells (iPSCs), are spherical structures with an inwards-facing apical membrane, and therefore not ideally suited for exposure studies that require exposure on the apical side in a barrier model configuration. Apical-out intestinal organoids can be achieved (Co et al. 2021); however, this would in turn complicate basolateral exposure and sampling. Moreover, the disassembly of organoids into monolayers causes the loss of self-organized cell patterns (Lewis et al. 2021). Unlike the primary tissue and adult stem cells, iPSC lines are commercially available and can be grown indefinitely. iPSC differentiation can be directed into two-dimensional IEC monolayers (Kabeya et al. 2020), which were recently optimized to study the effects of per- and polyfluoroalkyl substances (PFAS) and heat-killed bacteria (Janssen et al. 2024a, b). The diverse cell population and consistent barrier integrity of the two-dimensional iPSC-derived IECs make it an interesting model to study inflammation-related perturbations of the human intestinal epithelium.

In the present study, the inflammatory responsiveness of intestinal epithelial cell layers of iPSC-derived IECs and differentiated Caco-2 cells in response to nigericin and LPS co-exposure was evaluated and compared. Inflammatory responses were assessed by measuring cytotoxicity, barrier integrity, mRNA expression, and secretion of relevant cytokines upon exposure.

## Methods

### Culture of hiPSC

Human iPSCs (CS83iCTR-33nxx) were obtained from Cedars-Sinai Medical Center’s David and Janet Polak Foundation Stem Cell Core Laboratory (CA, United States) and cultured in a standard gas atmosphere with 95% humidity and 5% CO_2_ at 37 °C under feeder-free conditions using Matrigel hESC-Qualified Matrix-coated six-well plates (Corning, NY, USA). The cells were routinely passaged using Gentle Cell Dissociation Reagent (GCDR; Stem Cell Technologies, Saint-Egrève, France) for 6 minutes (min) at room temperature. mTeSR™ Plus medium (Stem Cell Technologies, Saint-Egrève, France) was changed every 2–3 days.

### hiPSC differentiation into intestinal epithelial cells

Intestinal epithelial cell induction was adapted from Janssen et al. (2024a, b). hiPSCs were dissociated into single cells with Accutase (Fisher Scientific, Landsmeer, Netherlands) for 6 min at 37 °C and 63,000 cells/cm^2^ were seeded in Matrigel hESC-Qualified Matrix-coated 24-well plates and subsequently incubated in mTeSR™ Plus medium with 10 μM Y-27632 (Stem Cell Technologies, Saint-Egrève, France) for 24 h. Definitive endoderm differentiation was subsequently induced using RPMI-1640 medium (Merck, Amsterdam, Netherlands) supplemented with 1% penicillin–streptomycin (10,000 U/mL) (Pen/Strep), 2 mM l-glutamine (Fisher Scientific, Landsmeer, Netherlands), 1% non-essential amino acids (NEAA), 2% B27 supplement minus vitamin A (Fisher Scientific, Landsmeer, Netherlands), 100 ng/mL Activin A (Cell guidance systems, Cambridge, United Kingdom), and 50 ng/mL bone morphogenetic protein 4 (BMP4, R&D Systems, Dublin, Ireland). On the second day, BMP4 was removed, and the medium was changed every day for 2 consecutive days. Intestinal stem cell induction was performed between days 3 to 7 in Dulbecco’s modified Eagle’s medium (DMEM)/nutrient mixture F-12 (F-12) medium (Fisher Scientific, Landsmeer, Netherlands) supplemented with 2% defined fetal bovine serum (dFBS; Cytiva, MA, United States), 1% Glutamax (Fisher Scientific, Landsmeer, Netherlands), and 250 ng/mL fibroblast growth factor 2 (FGF2, R&D Systems, Dublin, Ireland). On day 7, the cells were dissociated using Accutase for 6 min at 37 °C. Millicell 24-well inserts with pore size of 3.0 µm were pre-coated with Matrigel Growth Factor Reduced Basement Membrane Matrix (Corning, NY, United States) and 225,000 cells/cm^2^ were seeded on the apical side of the Matrigel-coated insert in intestinal cell differentiation medium (Advanced DMEM (Fisher Scientific, Landsmeer, Netherlands) supplemented with 2% dFBS, 2% B27 supplement minus vitamin A, 2% HepExtend supplement, 1% N2 supplement, 1% NEAA, 1% Pen/Strep, 2 mM l-glutamine, 20 ng/mL epidermal growth factor (EGF; R&D Systems, Dublin, Ireland), and 3 µM Forskolin (Stem Cell Technologies, Saint-Egrève, France). 10 μM Y-27632 was added during the first 3 days. The choice for Millicell inserts was based on the distributor’s availability and its compatibility with organ-on-chip devices. The plates were then shaken at 60 rpm on a SH-200D-O Mini Orbit Shaker (Cole-Parmer, Wertheim, Germany) with medium changes every 2–3 days. Intestinal cell differentiation medium was supplemented from day 14 onwards with the small molecules 5 µM 5-aza-2′-deoxycitidine (Fisher Scientific, Landsmeer, Netherlands), 20 µM PD98059 (Stem Cell Technologies, Saint-Egrève, France) and 0.5 µM A-83-01 (Stem Cell Technologies, Saint-Egrève, France) until 26–28 days of differentiation. Cell culture was performed at 37 °C with 5% CO_2_ for all differentiation steps.

### Gene expression analysis

**Fig. 1:**
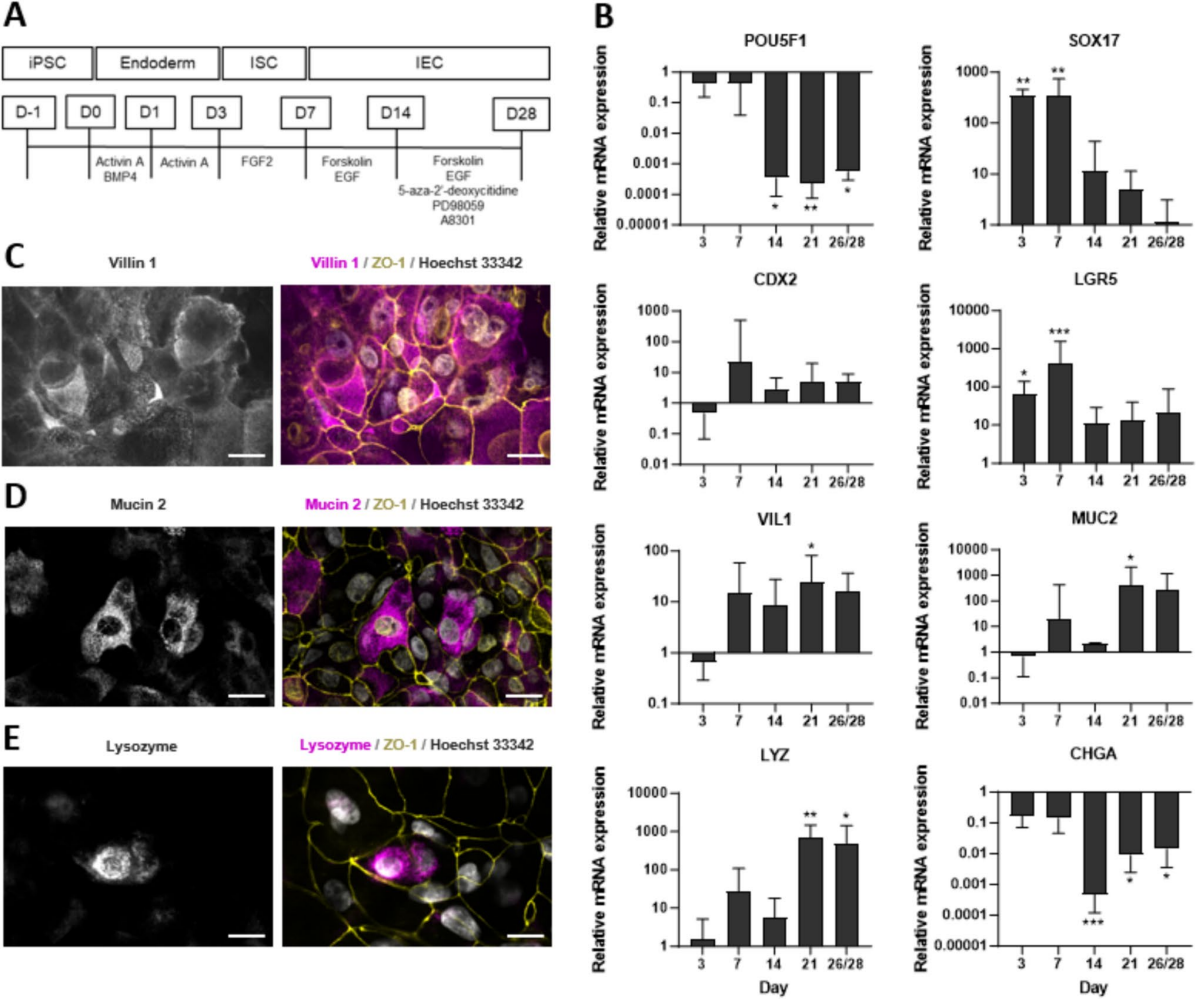
Differentiation and characterization of two-dimensional iPSC-derived IEC layers. **A** Schematic outline of the differentiation process of iPSCs towards IEC layers via treatment with the indicated compounds. **B** mRNA expression levels of POU5F1, SOX17, CDX2, LGR5, VIL1, MUC2, LYZ, and CHGA were assessed via RT-qPCR on days 3, 7, 14, 21, and 26/28 (one of the four replicates was differentiated until day 26 instead of 28). Samples were normalized to ACTIN and GAPDH expression and presented as a fold change of the expression on day 0. Expression levels are presented as the mean ± SD (*N* = 4) and compared to day 0 using the Kruskal–Wallis test followed by Dunn’s multiple comparisons test. Differences with *p* < 0.05 were considered significant (* = *p* < 0.05, ** = *p* < 0.01, *** = *p* < 0.001, **** = *p* < 0.0001). **C**–**E** Immunofluorescent staining of iPSC-derived IEC layers on day 28 for the proteins Villin 1, Mucin 2, and Lysozyme. Nuclei were stained with Hoechst 33342, and tight junction networks with anti-ZO-1 antibodies to visualize the overall morphology of the cell layers. Images were taken at 60 × magnification, scale bar represents 20 µm

Total RNA was extracted using the RNeasy Micro kit (Qiagen, Venlo, Netherlands), according to the manufacturer’s instructions. RNA concentration was determined using a NanoDrop OneC Microvolume UV–Vis spectrophotometer (Fisher Scientific, Landsmeer, Netherlands) and adjusted to 30 ng/μL in DEPC-treated ultrapure water. Reverse transcription was performed using the Quantitect Reverse Transcriptase kit (Qiagen, Venlo, Netherlands) and an iCycler (Bio-Rad, Veenendaal, Netherlands) according to the manufacturer’s instructions. Real-time quantitative polymerase chain reaction (RT-qPCR) experiments were carried out on a Rotor-Gene Q (Qiagen, Venlo, Netherlands) using the Rotor-Gene SYBR green PCR kit (Qiagen, Venlo, Netherlands). CT values above 35 were excluded from the results. The primers used are listed in Table S2. The efficiency of the primers was checked prior to sample measurement. Values were quantified using the comparative threshold cycle method. Target gene mRNA expression was normalized to the average of *ACTIN* and *GAPDH* expression per sample and the negative control per exposure group. Fold changes were calculated based on day 0 for Fig. 1 and the negative control for Fig. 3.

### Immunocytochemistry

The cells were fixed with 3.7% paraformaldehyde (Merck, Amsterdam, Netherlands) in phosphate-buffered saline (PBS; Fisher Scientific, Landsmeer, Netherlands) at room temperature for 15 min, washed twice, and stored in MilliQ water at 4 °C. Cells were permeabilized with 0.3% Triton^®^ X-100 (Merck, Amsterdam, Netherlands) for 10 min and blocked with 2% fetal bovine serum (FBS), 2% bovine serum albumin (BSA), and 0.1% Tween20 (Merck, Amsterdam, Netherlands) in PBS for 30 min. The membranes from the Millicell inserts were subsequently removed with a scalpel and incubated with primary antibodies for 2 h at RT. Thereafter, the cells were incubated with secondary antibodies and Hoechst 33342 (Fisher Scientific, Landsmeer, Netherlands) for 30 min. The antibodies, along with all other materials used, are listed in Table S1. The inserts were placed on a microscopic slide with cells facing upwards and covered with a drop of Prolong Gold antifade mountant (Fisher Scientific, Landsmeer, Netherlands) and a cover slip. Images were captured with a re-scan confocal microscope (Confocal.nl, Amsterdam, Netherlands) using a 60 × magnification objective and Z-stack acquisition.

### Culture of Caco-2

Caco-2 cells (HTB-37) were obtained from ATCC (VA, United States) and passage 15–30 were used. Caco-2 cells were cultured in Dulbecco’s modified Eagle’s medium (DMEM) with high glucose, l-glutamine, and sodium pyruvate (Capricorn Scientific, Eborfergrund, Germany), supplemented with 20% FBS (Fisher Scientific, Landsmeer, Netherlands), 1% NEAA (LifeTech, 11140050), 1% Pen/Strep, and 1% GlutaMAX (Fisher Scientific, Landsmeer, Netherlands). Caco-2 cells were trypsinized with 0.5% trypsin–EDTA (Fisher Scientific, Landsmeer, Netherlands) for 6 min and 200,000 cells/cm^2^ were seeded on the apical side of Millicell 24-well inserts (Merck Millipore, Darmstadt, Germany). The cells were incubated for 21 days at 37 °C with 5% CO_2_ to differentiate into enterocyte-like epithelial cells. The medium was changed every 2–3 days.

### Exposure to microbial toxins

After cultivating the hiPSC cells for 26–28 days and the Caco-2 cells for 21 days, the apical or basolateral medium was removed and replaced with fresh medium containing 100 ng/mL LPS from *Escherichia coli* O111:B4 (Merck, Amsterdam, Netherlands) together with 0–100 µM of nigericin sodium salt (Merck, Amsterdam, Netherlands) on either the apical or basolateral side for 24 h. The supernatant was collected from the apical and basolateral compartments and stored separately at − 20 °C.

### Transepithelial electrical resistance measurements

Transepithelial electrical resistance (TEER) was measured using the EVOM3 Manual Epithelial Volt Ohm Meter (World Precision Instruments, Friedberg, Germany) and STX4 EVOM™ Electrode (World Precision Instruments, Friedberg, Germany). TEER was measured according to the manufacturer’s instructions. The samples were measured before medium changes (every 2–3 days) and kept on a heated flask during measurements. Cell-free Matrigel-coated inserts were used as blank values. TEER values were normalized by subtracting the blank and multiplying by the insert surface area of 0.6 cm^2^. Monolayers of differentiated Caco-2 cells and iPSC-derived IECs were considered of acceptable quality for the exposure experiments if the normalized TEER values were higher than 150 Ω*cm^2^ and 400 Ω*cm^2^, respectively.

### Lactate dehydrogenase (LDH) assay

LDH reaction solution (homebrew) was prepared in advance by combining 50 µL lithium l-lactate (204 mM), 46 µL nicotinamide adenine dinucleotide sodium salt (5 mM), 2 µL iodonitrotetrazolium chloride (65 mM), and 2 µL phenazine methosulfate from Merck (29 mM) (Amsterdam, Netherlands) with 50 µL Tris buffer (200 mM, pH 8) (Trizma hydrochloride; Merck, Amsterdam, Netherlands, Trizma base; Merck, Amsterdam, Netherlands). After 24 h of LPS and nigericin exposure, 50 µL of apical and basolateral supernatant of each insert was transferred to a 96-well plate. 150 µL of LDH reaction solution was added to the supernatants and incubated at 37 °C and 5% CO_2_ for 5–15 min. The reaction was stopped by adding 50 µL of 1 M H_2_SO_4_ (Honeywell International Inc., Charlotte, NC, United States). Absorbance was measured using a SpectraMax iD3 plate reader and SoftMax Pro 7.1 (Molecular Devices, SJ, United States) at 490 and 680 nm. Data are presented as A_490nm_–A_680nm_ as fold change of the untreated control. Cells subjected to 0.5% Triton for 15 min were used as a positive control for cell death.

### Enzyme-linked immunosorbent assay (ELISA)

The release of pro-inflammatory cytokines in the apical medium following exposure to bacterial toxins was analyzed using ELISA kits (R&D Systems, Dublin, Ireland) for human Interleukin (IL)−6, IL-8, and tumor necrosis factor-alpha (TNF-α) as described elsewhere (Busch et al. 2021). The supernatants were diluted if necessary.

### Alcian blue/PAS staining

Cells on Millicell inserts were fixed as described in the Immunocytochemistry section. Cells were pre-treated with 3% acetic acid (Merck, Amsterdam, Netherlands) for 3 min, followed by 1% Alcian Blue (Merck, Amsterdam, Netherlands) in 3% acetic acid for 30 min. Cells were subsequently washed thrice with MilliQ water and pre-treated with 1% periodic acid solution (Merck, Amsterdam, Netherlands) for 10 min. After washing three times with MilliQ, Schiff’s reagent (Merck, Amsterdam, Netherlands) was added to each sample for 15 min, followed by three times sulfite water for 2 min and two times MilliQ wash. The insert was removed and mounted on a microscopic slide as described in the Immunocytochemistry section.

### Statistics and data analysis

**Fig. 2:**
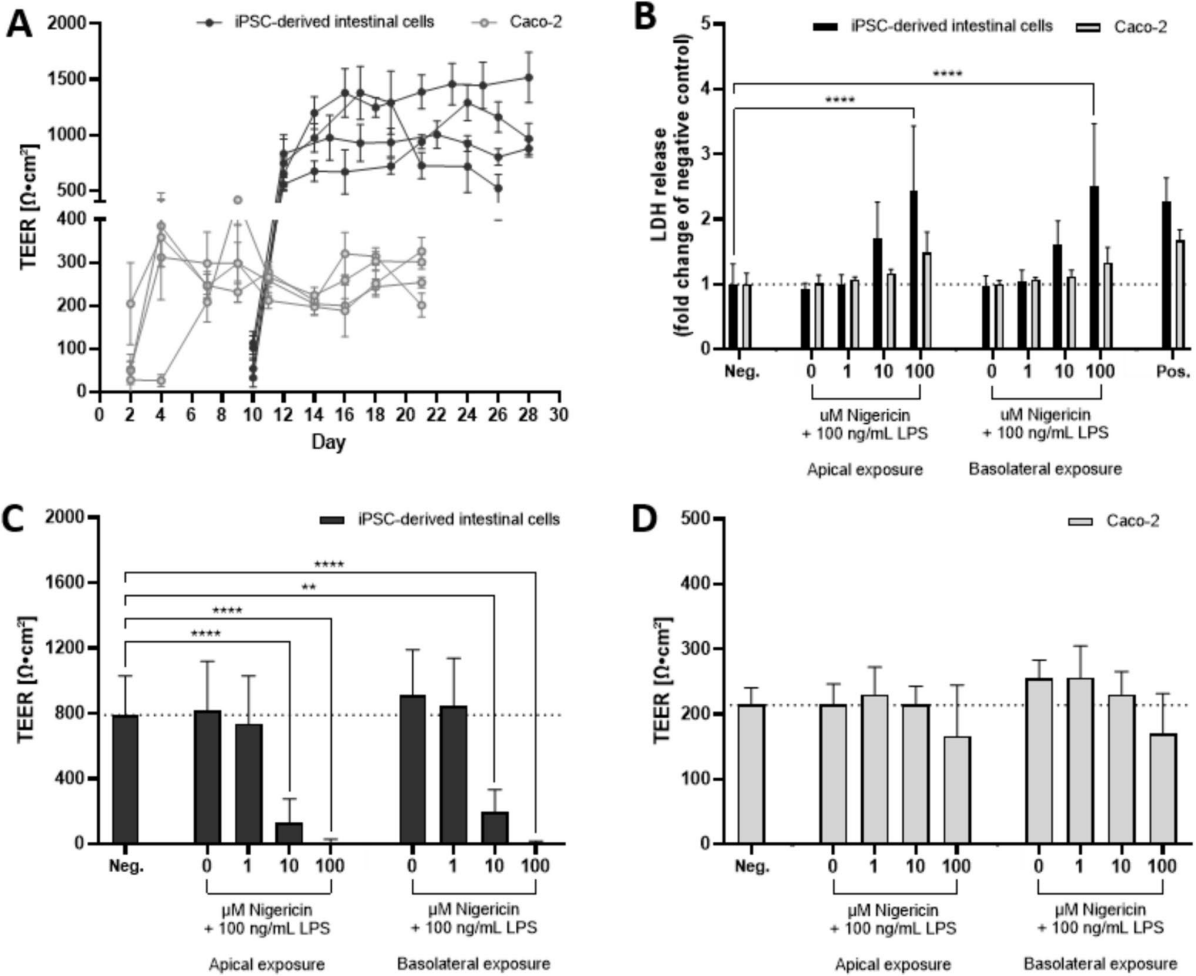
Barrier integrity and cytotoxicity in two-dimensional iPSC-derived IEC- and Caco-2 cell layers. **A** TEER measurements in the cell layers of iPSC-derived IECs (black) and Caco-2 cells (grey). iPSC-derived IECs were first differentiated in 24-well plates and subsequently reseeded on Matrigel-coated Millicell inserts on day 7. Caco-2 cells were seeded directly onto inserts and differentiated for 21 days. Each line represents a single independent experiment (*N* = 4), with each data point on the line representing the average of all technical replicates within the experiment. **B** LDH levels in the apical supernatant of iPSC-derived intestinal cells (black bars) and Caco-2 cells (grey bars) after 24 h exposure to LPS plus 0–100 µM nigericin on the apical or basolateral side. Data are expressed as the fold change compared to the negative control (medium only; dotted line) as the mean ± SD (*N* = 4) and compared to the negative control using a one-way ANOVA followed by Dunnett’s multiple comparisons test. **C**, **D** TEER after 24 h exposure to LPS plus 0–100 µM nigericin on the apical or basolateral side. The dotted line indicates the average TEER value for the negative control of both iPSC-derived IECs and Caco-2 cells, depicted as mean ± SD (*N* = 4). Differences with *p* < 0.05 were considered significant (** = *p* < 0.01, *** = *p* < 0.001, **** = *p* < 0.0001)

**Fig. 3:**
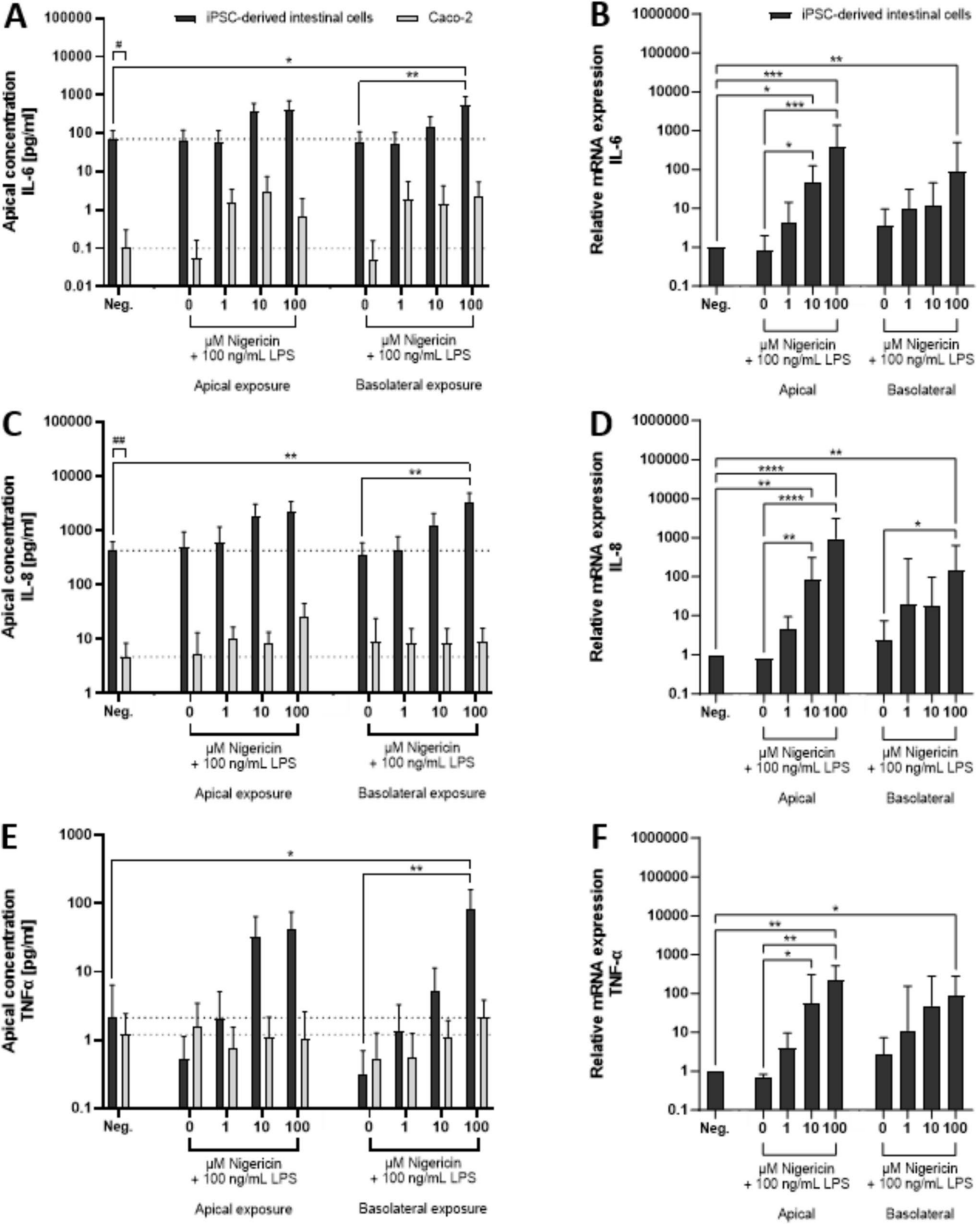
Pro-inflammatory response after exposure to LPS and nigericin in iPSC-derived IECs and Caco-2 models. **A**, **C**, **E** Apical secretion of IL-6, IL-8, and TNF-α from iPSC-derived intestinal cells (black bars) and differentiated Caco-2 cells (gray bars) after 24 h of exposure to 100 ng/mL LPS with 0–100 µM nigericin. The dotted line indicates the average cytokine concentration in the negative control of both iPSC-derived IECs and Caco-2 cells. **B**, **D**, **F** Relative mRNA expression levels of IL-6, IL-8, and TNF-α in IPSC-derived IECs after 24 h of exposure to 100 ng/mL LPS with 0–100 µM nigericin. Expression levels were determined by RT-qPCR and normalized to GAPDH and ACTIN and expressed as fold change of the negative control (Neg.) per cytokine. Expression levels are presented as the mean ± SD (*N* = 4) and compared to the negative control using a one-way ANOVA followed by Dunnett’s multiple comparisons test. Differences with *p* < 0.05 after multiple testing corrections were considered statistically significant (*p < 0.05, **p < 0.01, ***p < 0.001, ****p < 0.0001). Differences between the cytokine concentration of the negative controls of Caco-2 and iPSC-derived IECs were tested with a student’s *t* test. Differences with *p* < 0.05 were considered statistically significant (^#^*p* < 0.05, ^##^*p* < 0.01)

Data sets were analyzed using GraphPad Prism software version 10 (GraphPad Software, CA, USA). Unless stated otherwise, values are expressed as the mean ± standard deviation (SD) of four independent experiments (*N* = 4) and three technical replicates in each experiment. Normal distribution in the data was tested with the Shapiro–Wilk test. Statistical analysis was performed using the Kruskal–Wallis test with Dunn’s multiple comparison test for the gene expression of differentiation makers in Fig. 1b and one-way analysis of variance (ANOVA), followed by Dunnett’s multiple comparisons test for TEER, LDH, and gene expression data in Figs. 2b–d and 3. Differences with *p* < 0.05 were considered significant (*p < 0.05, **p < 0.01, ***p < 0.001, ****p < 0.0001). In Fig. 3, differences between the cytokine expression of the negative controls were tested with unpaired *t* test and considered significant if *p* < 0.05 (^#^*p* < 0.05, ^##^*p* < 0.01, ^###^*p* < 0.001, ^####^*p* < 0.0001).

## Results

### Intestinal epithelial-like cell layer differentiation

iPSCs were differentiated into IECs using a three-step differentiation protocol (Fig. 1a). In the current protocol, intestinal stem cells were replated onto Matrigel-coated Millicell inserts to obtain a barrier model separating the apical and basolateral compartments. To confirm the path of cell differentiation, expression of cell-specific genes was analyzed at critical time points during differentiation (Fig. 1b). In accordance with our expectations, mRNA expression of the stem cell pluripotency marker *POU5F1* significantly decreased (as seen on days 14, 21 and 28) compared to expression levels detected in undifferentiated iPSCs on day 0 (Fig. 1b). Exposure of iPSCs to Activin A and BMP4 from day 1 onwards significantly increased the mRNA expression of *SOX17* on day 3 and 7 (Fig. 1b), which is a marker indicating differentiation towards an endodermal cell fate. Differentiation towards intestinal stem cells was marked by its peak *LGR5* expression on day 7 (Fig. 1b). The increased mRNA expression of *Villin 1* (*VIL1*) (Fig. 1b) between days 7 and 28 and the resulting *VIL1* protein expression at day 28 (Fig. 1c) suggests the differentiation into enterocyte-like cells. The presence of MUC2-positive cells was confirmed by the increasing mRNA expression of *MUC2* and immunocytochemistry at the later time points (Fig. 1b, d). Histochemical analysis revealed an increasing intensity of blue and magenta Alcian Blue/PAS-positive cell clusters on top of the IEC cell monolayers, further indicating the presence of acidic and neutral mucus in the cell layers (Fig. S1). In addition, increasing *Lysozyme* (*LYZ*) mRNA expression throughout differentiation was indicative of the presence of Paneth cells (Fig. 1b), which was confirmed by immunocytochemistry (Fig. 1e). No evidence was found of iPSC differentiation towards enteroendocrine cells, as the immunostaining for the commonly used endocrine marker chromogranin A (CHGA) did not show positive cells (data not shown), and gene expression for *CHGA* in IECs significantly decreased relative to iPSCs at day 0 from day 14 onwards (Fig. 1b).

### Intestinal epithelial cell layer barrier integrity

The barrier integrity of iPSC-derived IEC layers and Caco-2 cell layers was assessed during differentiation using intermittent TEER measurements every 2–3 days. TEER remained within acceptable boundaries from day 4 for Caco-2 and day 14 for iPSC-derived IECs, respectively, averaging ~ 300 Ω*cm^2^ and ~ 1000 Ω*cm^2^ (Fig. 2a). Although differences in TEER values between the two models were observed, three technical replicates within each independent experiment displayed comparable TEER values (Fig. 2a).

Before analysis of the inflammatory responsiveness of the two cell models, LPS and nigericin were added to assess their potency to induce cytotoxicity and barrier disruption. Cytotoxicity was measured using the LDH assay on supernatants from both the apical (Fig. 2b) and basolateral side (Fig. S2) after apical or basolateral exposure for 24 h. The highest concentration of nigericin (100 µM) in combination with 100 ng/mL LPS-induced significantly increased LDH release from iPSC-derived IEC layers into the apical compartment, but not from differentiated Caco-2 cell layers, irrespective of apical or basolateral exposure (Fig. 2b). The TEER values of iPSC-derived IEC layers decreased in a concentration-dependent manner, which reached statistical significance upon exposure to 10 µM nigericin in combination with 100 ng/mL LPS when exposed either to the apical or basolateral side. No effects on TEER were observed in the cell layers of differentiated Caco-2 cells (Fig. 2d). We noticed a slight decrease in baseline TEER post-exposure (~ 1000 to ~ 800 Ohm*cm^2^ in iPSCs and ~ 300 to ~ 200 Ohm*cm^2^ in Caco-2, see Fig. 2a, c, d), which is likely an effect of measuring TEER 24 h after medium change in the exposure experiment, as opposed to measuring 48–72 h after medium change during the differentiation period.

### LPS and nigericin-induced pro-inflammatory cytokine mRNA expression and secretion

Upon 24 h of co-exposure to 100 ng/mL LPS with 0–100 µM nigericin, the secretion of pro-inflammatory cytokines IL-6, IL-8, and TNF-α by iPSC-derived IECs and Caco-2 cells was determined. Cell model-dependent differences were observed in the secretion of pro-inflammatory cytokines. Exposure of iPSC-derived IEC monolayers to increasing concentrations of nigericin in the presence of 100 ng/mL LPS showed a trend of increased release of IL-6, IL-8, and TNF-α (Fig. 3a, c, e), whereas basolateral exposure to the highest concentration of nigericin caused a statistically significant effect. No significant differences in the secretion of IL-6, IL-8, and TNF-α in the differentiated Caco-2 cells were observed compared to the negative control (Fig. 3b, d, f). Of note, the basal secretion of IL-6 and IL-8 was significantly higher in the iPSC-derived IECs compared to the secretion by differentiated Caco-2 cells (Fig. 3a, c).

To determine whether transcription of *IL-6*, *IL-8*, and *TNF-α* genes in iPSC-derived IECs was upregulated upon LPS and nigericin exposure, the mRNA expression was determined using RT-qPCR. None of the cytokine mRNA expression levels were affected by LPS exposure alone, while significantly increased *IL-6*, *IL-8* and *TNF-α* mRNA expression was observed when cells were co-exposed to LPS and nigericin at 10 µM apically or 100 µM basolaterally (Fig. 3b, d, f).

## Discussion

The current study compared the capacity of two different intestinal in vitro models to emulate the human intestinal inflammatory responsiveness and subsequent effects on barrier integrity. iPSC-derived IECs were found to be more responsive to LPS and nigericin exposure than differentiated Caco-2 cells. This makes the iPSC-derived IECs a more suitable model to study inflammation-related perturbations of the human intestinal epithelium in vitro than Caco-2 cells.

Previous studies have shown that iPSCs can differentiate into IECs (Janssen et al. 2024a, b; Kabeya et al. 2020). The protocols used in the aforementioned studies make use of forskolin for IEC differentiation. Yet this might limit the applicability of the derived IECs, as forskolin has been shown to inhibit the integral pathways in the inflammation pathway (Brodsky et al. 1998; Chen et al. 2019; Yan et al. 2015). Therefore, the forskolin concentration used for IEC differentiation in the current study was reduced from 30 to 3 µM to have a negligible effect on inflammation pathways (Yan et al. 2015). In addition, iPSC-derived cells typically have an immature or fetal-like phenotype (Volpato and Webber 2020), while cells exposed to continuous flow and shear stress were found to be more mature (Deguchi et al. 2024). Thus, orbital shaking was used to induce shear stress on both the apical and basolateral sides to promote cellular maturation. Indeed, subtle modifications in the cocktail of small molecules used during differentiation can alter the ratios between intestinal epithelial cell type populations in vitro (Beumer and Clevers 2021; Efe and Ding 2011). The expression of hallmark differentiation markers was confirmed to assess the progression of each differentiation stage during differentiation and expansion. Furthermore, cell-type-specific immunostaining markers indicated the presence of enterocytes, goblet cells and Paneth cells on day 28 of culture. A time-dependent decrease of *POU5F1* expression indicates loss of pluripotency (Matin et al. 2004), while *LGR5* is still expressed at the end of the differentiation procedure, confirming the presence of non-terminally differentiated intestinal stem cells in the cell population (Koo and Clevers 2014). The upregulation of *SOX17* on day 3 and 7 indicates Activin A-mediated differentiation towards definitive endoderm as an intermediate step, while endodermal identity is lost again at the end of differentiation (day 28), as seen by a downregulation of *SOX17* (Wang et al. 2011). Further, the presence of enterocytes, goblet cells, and Paneth cells was confirmed using gene expression of *VIL1*, *MUC2*, and *LYZ*, respectively, and immunostaining of the associated proteins. The expression of *CHGA*, a marker of enteroendocrine cells that are occasionally observed in iPSC-derived intestinal models (Janssen et al. 2021), was not observed. Overall, these gene expression patterns are in line with previous characterization studies of iPSC-derived IECs (Janssen et al. 2024a, b).

Exposure of iPSC-derived IECs to LPS in combination with nigericin significantly reduced TEER upon increasing nigericin concentrations, irrespective of apical or basolateral exposure to these compounds. An increase in the release of LDH by the iPSC-derived IECs was also observed at the highest compound concentrations tested, indicating major necrotic cell death through membrane damage. Nigericin is a natural antibiotic produced by *Streptomyces hygroscopicus*, which has been used as a costimulatory inducer of intestinal inflammation (Antonopoulos et al. 2015; Lei-Leston et al. 2017; Perregaux and Gabel 1994). Nigericin acts as an ionophore, allowing in- and efflux of several types of ions, including H^+^ and K^+^, leading to changes in pH gradients and mitochondrial membrane potential (Rozario et al. 2024). Furthermore, nigericin is a potent activator of inflammasomes, which, once activated, lead to the release of pro-inflammatory cytokines and possibly pyroptosis, an inflammatory form of lytic cell death. Cell death upon nigericin treatment has been reported in several cell models (Cao et al. 2022; He et al. 2015; Wu et al. 2023). In Caco-2 cells, LPS and nigericin only induced very minor, non-significant effects on TEER and LDH release, hinting either towards higher robustness of these cells or the previously reported lack of crucial receptors/pathways that are involved in the response towards nigericin (Furrie et al. 2005).

Although LPS is commonly reported as a potent activator of multiple pro-inflammatory pathways in other in vitro models (Sharif et al. 2007), 100 ng/mL LPS alone did not induce any pro-inflammatory effect in the iPSC-derived IECs. Interestingly, a combination with at least 10 µM nigericin seems to be sufficient to result in detectable changes in markers indicative of intestinal inflammation, such as an impaired barrier, cell death, and release of the pro-inflammatory cytokines IL-6, IL-8, and TNF-α. IL-6 has been described to be essential in bacterial–epithelial cross-talk in the gastrointestinal tract (Guo et al. 2021). IL-8 is a chemoattractant largely produced by intestinal epithelial cells to recruit immune cells in response to bacterial invasion (Li et al. 1998). The cytokine TNF-α has been described to have diverse IEC effects, such as modulation of mucus secretion, tight junction control, and cell death (Leppkes et al. 2014). Overall, the response of the iPSC-derived IECs is a plausible reaction towards the bacterial toxins LPS and nigericin and resembles an in vivo reaction to bacterial invasion of the gut.

Furthermore, our study assessed whether the inflammatory reaction of iPSC-derived IECs towards LPS and nigericin differs between apical and basolateral exposure. The presence and location of specific TLRs on IECs can vary based on tissue region and disease state (Yu and Gao 2015), while TLRs are generally expressed on both apical and basolateral membranes of IECs (Bruning et al. 2021). Despite subtle differences in the cytokine expression and release after apical and basolateral exposure, we cannot conclude any side to be more susceptible towards bacterial toxins. Both scenarios are physiologically relevant, as (fragments of) bacteria might either breach the intestinal mucus layer and interact with the apical membrane of the intestinal epithelium, or breach the intestinal epithelial layer itself and interact with receptors on the basolateral side. In contrast, no effects on cytokine secretion were observed in differentiated Caco-2 cells. It has been previously described that Caco-2 cells show very limited responses towards inflammatory stimuli such as LPS (Kämpfer et al. 2017), which in part can be attributed to a lack of *TLR4* expression (Böcker et al. 2003). However, nigericin exposure did not cause any effects in Caco-2 cells as well, suggesting a dysfunction of a nigericin-associated pathway. Overall, our results demonstrate immunocompetence of iPSC-derived IECs towards the microbial toxins LPS and nigericin, even without a specific immunocompetent cell type present. Notably, the basal mRNA expression of *IL-6*, *IL-8*, and *TNF-α*, and their respective secretion, was significantly elevated in the cell layers of iPSC-derived IECs compared to that in Caco-2 cell layers, indicating a constant state of low-level inflammation in iPSC-derived IECs without additional stimuli. A possible explanation could be the activation of transcription factor STAT3, which is involved in IL-6 production, but also in the establishment of pluripotency in iPSCs (Wang et al. 2018). iPSC-derived cells often represent an immature phenotype compared to cells directly collected from in vivo material, as iPSC-IECs are not exposed to the physiologically relevant tissue microenvironment that might play a major role in fine-tuning gene expression patterns (Cerneckis et al. 2024). The iPSC-IECs immature and more proliferative state could explain its significantly higher cytokine expression compared to Caco-2.

A true recapitulation of intestinal immune responses involves the interplay between epithelial and immune cells and thus requires the incorporation of intestinal tissue-residing immune cells in future research. Tissue-resident immune cells, most notably macrophages and dendritic cells, underlie the intestinal epithelial cells and induce hyporesponsiveness to bacteria (Galli and Saleh 2021; Stagg et al. 2003). If the intestinal barrier is breached, tissue-resident immune cells release cytokines and chemokines to induce mucosal inflammation. Several groups have reported this interplay between the epithelial lining and immune cells in the intestine, often using the THP-1 cell line (Kämpfer et al. 2017; Macedo et al. 2023). To fully recapitulate the intestinal inflammatory response induced by luminal content, a co-culture of epithelial and tissue-resident immune cells [e.g., macrophages (Cao et al. 2020)] must be further explored, potentially derived from the same donor.

Moreover, initial results from multicenter studies, using complex iPSC models for drug screening, showed acceptable variability, overcoming a main hurdle for the broader acceptance of the outcome of iPSC-based studies (Pang et al. 2024). Recent examples show that iPSC-based cell models have successfully studied the transport and effects of PFAS (Janssen et al. 2024a, b), paving the way for other transport studies using iPSC-based models (Burnett et al. 2021; Ford et al. 2024). In addition, iPSC-derived models have the potential to study individual donor differences, which are particularly important in the context of disease modeling, such as IBD. Alleles that increase disease susceptibility often show low penetrance in individuals carrying them, indicating that IBD is primarily caused by the interplay between host factors and environmental influences (Maloy and Powrie 2011). To distinguish between genetic predisposition and overlapping pathogenic exposure, cellular models should include donors with disease-susceptibility alleles for disease modeling and risk analysis. Cells from patients with IBD could be collected using non-invasive methods and transformed into iPSCs, which could then be differentiated into iPSC-derived intestinal cells, similar to how it was demonstrated for iPSCs derived from osteoarthritis patients (Castro-Viñuelas et al. 2020). The iPSC differentiation protocol and readout methods described in the present study could aid in the development of such personalized models that emulate the inflammation-related perturbations and subsequent treatment options.

## Conclusion

Overall, our study showed that iPSC-derived IEC layers are a suitable immunocompetent model of the intestinal epithelial cell layer, capable of emulating pro-inflammatory responses towards microbial toxins. This model could be further developed and used to emulate inflammation-related perturbations of the human intestinal epithelium in vitro, stemming from drugs, chemicals, particulate matter, dietary components, or disease susceptibility.

## Supplementary Information

Below is the link to the electronic supplementary material.

Supplementary file 1 (DOCX 1446 kb)

## Data Availability

Data supporting the findings of this study are available from the corresponding author upon request.

## Abbreviations

ANOVA: Analysis of variance
BMP4: Bone morphogenetic protein 4
BSA: Bovine serum albumin
CHGA: Chromogranin A
DMEM: Dulbecco’s modified Eagle’s medium
dFBS: Defined fetal bovine serum
EGF: Epidermal growth factor
F-12: Nutrient mixture F-12
FBS: Fetal bovine serum
FGF2: Fibroblast growth factor 2
GCDR: Gentle cell dissociation reagent
H: Hours
IBD: Inflammatory bowel disease
IEC: Intestinal epithelial cells
IL: Interleukin
iPSC: Induced pluripotent stem cells
LDH: Lactate dehydrogenase
LPS: Lipopolysaccharides
LYZ: Lysozyme
min: Minutes
MUC2: Mucin 2
NEAA: Non-essential amino acids
PBS: Phosphate-buffered saline
Pen/Strep: Penicillin–streptomycin
PFAS: Per- and polyfluoroalkyl substances
RT-qPCR: Real-time quantitative polymerase chain reaction
SD: Standard deviation
TEER: Transepithelial electrical resistance
TLR: Toll-like receptors
TNF-α: Tumor necrosis factor-alpha
VIL1: Villin 1
ZO-1: Zonula occludens-1
