# Supplementary material for: Pro-inflammatory response of human iPSC-derived intestinal epithelial monolayers towards microbial toxins LPS and nigericin
Source: Arch Toxicol. 2025 Oct 8;100(2):695–708. doi: 10.1007/s00204-025-04215-9 (PMC12886239; doi:10.1007/s00204-025-04215-9)
Supplement: Supplementary file 1 — (DOCX 1446 kb) [file 204_2025_4215_MOESM1_ESM.docx]

## **Supplementary Material**

**Table S1** – Key resources

| **REAGENT or RESOURCE** | **SOURCE** | **IDENTIFIER** |
| --- | --- | --- |
| **Antibodies** | | |
| Anti-Villin antibody for Immunofluorescence (1:200) | Santa Cruz Biotechnology | Cat# sc-58897  RRID: AB_2304475 |
| Anti-MUC2 for immunofluorescence (1:100) | Santa Cruz Biotechnology | Cat# sc-7314  RRID: AB_627970 |
| Anti-Lyzozyme for immunofluorescence (1:100) | DAKO | Cat# A009902-2  RRID: AB_2341230 |
| Anti-ZO-1 for immunofluorescence (1:200) – used in combination with Anti-Villin and Anti-MUC2. | Fisher Scientific | Cat# PA585256  RRID: AB_2792399 |
| ZO-1 Monoclonal Antibody (ZO1-1A12), Alexa Fluor™ 594 (1:200) – used in combination with Anti-Lyzozyme. | Fisher Scientific | Cat# 339194  RRID: AB_2532188 |
| Goat anti-Mouse IgG (H+L) Highly Cross-Adsorbed Secondary Antibody, Alexa Fluor™ Plus 488 (1:250) | Fisher Scientific | Cat# A32723  RRID: AB_2633275 |
| Donkey anti-Rabbit IgG (H+L) Highly Cross-Adsorbed Secondary Antibody, Alexa Fluor™ 647 (1:250) | Fisher Scientific | Cat# A-31573  RRID: AB_2536183 |
| **Chemicals, Peptides, and Recombinant Proteins** | | |
| Matrigel hESC-Qualified Matrix | Corning | 354277 |
| Matrigel Growth Factor Reduced | Corning | 354230 |
| mTeSR plus | Stem cell technologies | 662640 |
| Gentle Cell Dissociation Reagent | Stem cell technologies | 100-0485 |
| Y-27632 | Stem cell technologies | 72304 |
| Recombinant Human Activin A | Cell guidance systems | GFH6-100 |
| Recombinant Human BMP-4 Protein | R&D systems | 314-BP-010/CF |
| Recombinant Human FGF2 Protein | R&D systems | 233-FB-025/CF |
| Recombinant Human EGF Protein | R&D systems | 236-EG-200/CF |
| Forskolin | Merck | F3917 |
| 5-aza-2’-deoxycytidine (5mM) | Merck | A3656 |
| A 83-01 | Stem cell technologies | 72022 |
| PD98059 | Stem cell technologies | 72174 |
| RPMI-1640 Medium | Merck | R8758 |
| DMEM/F-12 with 15 mM HEPES | Stem cell technologies | 36254 |
| Advanced DMEM/F-12 | Fisher Scientific | 12634010 |
| GlutaMAX™ Supplement | Fisher Scientific | 35050038 |
| B-27™ Supplement (50X), minus vitamin A | Fisher Scientific | 12587010 |
| N-2 Supplement | Fisher Scientific | 17502048 |
| HepExtend™ Supplement | Fisher Scientific | A2737501 |
| Accutase™ Cell Dissociation Reagent | Fisher Scientific | A1110501 |
| L-Glutamine (200 mM) | Fisher Scientific | 25030024 |
| Penicillin and Streptomycin (5,000 U/mL) | Fisher Scientific | 15070063 |
| Triton X-100 | Merck | T8787 |
| Tween20 | Merck | P9416 |
| Bovine Serum Albumin | Merck | A2153 |
| Lithium L-lactate | Merck | L2250 |
| β-Nicotinamide adenine dinucleotide sodium salt | Merck | N0632 |
| Iodonitrotetrazolium chloride | Merck | I8377 |
| Phenazine methosulfate | Merck | P9625 |
| Trizma hydrochloride | Merck | T3253 |
| Trizma base | Merck | T4661 |
| Sulfuric acid solution | Honeywell International Inc. | 84741 |
| Lipopolysaccharides from *Escherichia coli* O111:B4 | Merck | L4391 |
| Nigericin | Merck | N7143 |
| Hoechst 33342 | Fisher Scientific | H3570 |
| Defined Fetal Bovine Serum | Hyclone | SH30070.01 |
| GAPDH primer | Qiagen | PPH00150F-200 |
| TNFα primer | Qiagen | PPH00341F-200 |
| Acetic acid | Merck | 695092 |
| Alcian Blue solution | Merck | B8438 |
| Periodic acid | Merck | P7875 |
| Schiff’s reagent | Merck | 1.09033.0500 |
| **Critical Commercial Assays** | | |
| RLT buffer | Qiagen | 79216 |
| RNeasy mini kit | Qiagen | 74106 |
| QuantiTect Reverse Transcription Kit | Qiagen | 205311 |
| Rotor‐Gene® SYBR® Green PCR kit | Qiagen | 204174 |
| ProLong™ Gold Antifade Mountant | Fisher Scientific | P36934 |
| Human IL-6 DuoSet ELISA | R&D systems | DY206 |
| Human IL-8/CXCL8 DuoSet ELISA | R&D systems | DY208 |
| Human TNF-alpha DuoSet ELISA | R&D systems | DY210 |
| **Experimental Models: Cell Lines** | | |
| Caco-2 cells | ATCC | HTB-37 |
| hiPSC CS83iCTR-33nxx | Cedars-Sinai Medical Center | CS83iCTR-33nxx |
| **Equipment** | | |
| Orbital Shaker | Fisher Scientific | 10309644 |
| Nanodrop™ | Fisher Scientific | 13-400-525 |
| Rotor‐Gene Q® | Qiagen | 9001862 |
| EVOM3 Trans Epithelial Electrical Resistance (TEER) meters | WPI | EVOM3 |
| Electrode blades for STX4 EVOM™ | WPI | STX4-BLADES |
| SpectraMax | Molecular Devices | iD3 |
| Re-scan confocal microscope | Confocal.nl | RCM1 |
| Inverted phase contrast microscope | Olympus | CKX53 |
| **Software and Algorithms** | | |
| Fiji-ImageJ | Schindelin et al., 2012 PMID: 22743772 | https://imagej.net/Fiji/Downloads |
| GraphPad Prism 10.2.3 | GraphPad | N/A |
| **Disposables** | | |
| Millicell Cell Culture Insert, 12 mm, polycarbonate, 3.0 µm | Millipore | PITP01250 |
| Microscope slide 26x76x1mm | Fisher Scientific | 15998086 |
| Microscope cover glass, 170 µm thick | Fisher Scientific | 10039670 |
| QiaShredder | Qiagen | 79656 |
| Nunc™ MaxiSorp™ ELISA Plates, Uncoated | Biolegend | 423501 |

**Table S2** – Primers used for RT-qPCR

| **Gene symbol** | **GeneBank Accession No.** | **Primer sequence (5’-3’)** | **PCR efficiency (%)** | **Regression coefficient (R2)** | **Source** |
| --- | --- | --- | --- | --- | --- |
| POU5F1 | NM_002701.6 | F: TGGAGTTTGTGCCAGGGTTT  R: TCACCTTCCCTCCAACCAGT | 106 | 0.995 | This paper |
| SOX17 | NM_022454.4 | F: GCAAGATGCTGGGCAAGTC  R: CTTGTAGTTGGGGTGGTCCTG | 92 | 0.990 | This paper |
| CDX2 | NM_001265.6 | F: CCAGCGGCGGAACCTGTG  R: GTCTTTCGTCCTGGTTTTCAC | 110 | 0.995 | (Janssen et al., 2021) |
| LGR5 | NM_003667.4 | F: GGAAATCATGCCTTACAGAGC  R: CACTCCAAATGCACAGCACTG | 109 | 0.995 | (Janssen et al., 2021) |
| LYZ | NM_000239.3 | F: CCCTGGTCAGCCTAGCACTC  R: CCTTGCCCTGGACCGTAACA | 97 | 0.998 | (Janssen et al., 2021) |
| VIL1 | NM_007127.3 | F: CGGAAAGCACCCGTATGGAG  R: CGTCCACCACGCCTACATAG | 109 | 0.997 | (Janssen et al., 2021) |
| MUC2 | NM002457.4 | F: AGAAGGCACCGTATATGACGAC  R: CAGCGTTACAGACACACTGCTC | 107 | 0.999 | (Janssen et al., 2021) |
| CHGA | NM_001275.4 | F: TCCGACACACTTTCCAAGCC  R: TTCTGCTGATGTGCCCTCTC | 99 | 0.997 | (Janssen et al., 2021) |
| IL6 | NM_000600.3 | F: AGCCACTCACCTCTTCAGAAC  R: GCCTCTTTGCTGCTTTCACAC | 105 | 0.992 | (Ching et al., 2013) |
| IL8 | NM_000584.2 | F: CTGATTTCTGCAGCTCTGTG  R: GGGTGGAAAGGTTTGGAGTATG | 103 | 0.997 | (Teodorowicz et al., 2017) |
| TNF-α | NM_000594 | PPH00341F-200 | - | - | Qiagen |
| GAPDH | NM_002046 | PPH00150F-200 | - | - | Qiagen |
| ACTB | NM_001101.5 | QT00095431 | - | - | Qiagen |

**
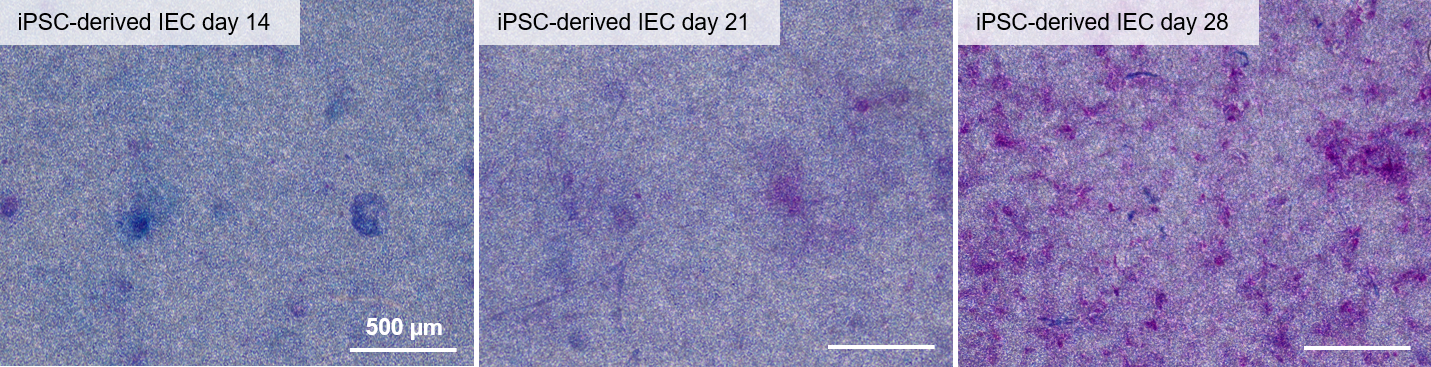
**

**Fig. S1** – Histochemical staining with Alcian blue and Periodic acid–Schiff reaction was performed on paraformaldehyde-fixed iPSC-derived intestinal-like epithelial cells for acidic (blue) and neutral (magenta) mucus, respectively. Images were acquired on an Olympus CKX53 inverted phase contrast microscope. Images were taken at 20x magnification, scalebar represents 500 µm.

**
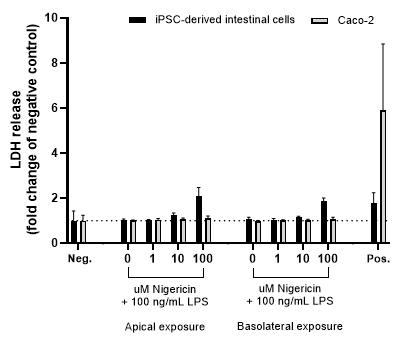
**

**Fig S2** – Basolateral LDH levels after exposure to LPS plus nigericin on the apical or basolateral sides. Data are expressed as the percentage change compared to the negative control as the mean ± SD (N=4).
